# Light-Driven Nanonetwork Assembly of Gold Nanoparticles via 3D Printing for Optical Sensors

**DOI:** 10.1021/acsanm.4c01673

**Published:** 2024-05-23

**Authors:** Arunachalam Ramanathan, Shuai Feng, Abhishek Saji Kumar, Sri Vaishnavi Thummalapalli, Martin Taylor Sobczak, Lindsay R. Bick, Kenan Song, Sui Yang

**Affiliations:** † Mechanical Engineering, College of Engineering, University of Georgia, 302 East Campus Road, Athens, 30602, United States; ‡ Materials Science and Engineering, School for Engineering of Matter, Transport and Energy (SEMTE), Ira A. Fulton Schools of Engineering, Arizona State University (ASU), Tempe, Arizona 85287, United States; § Associate Professor of Mechanical Engineering, School of Environmental, Civil, Agricultural, and Mechanical Engineering (ECAM), College of Engineering, University of Georgia, Athens, Georgia 30602, United States; ∥ Adjunct Professor, The School of Manufacturing Systems and Networks (MSN), Ira Fulton School of Engineering, Arizona State University, Mesa, Arizona 85212, United States; ⊥ Assistant Professor of Material Science and Engineering, School for Engineering of Matter, Transport and Energy (SEMTE), Ira A. Fulton Schools of Engineering, Arizona State University (ASU), Tempe, Arizona 85287, United States

**Keywords:** 3D printing, nanomanufacturing, self-assembly, nanoparticle, plasmonic resonance

## Abstract

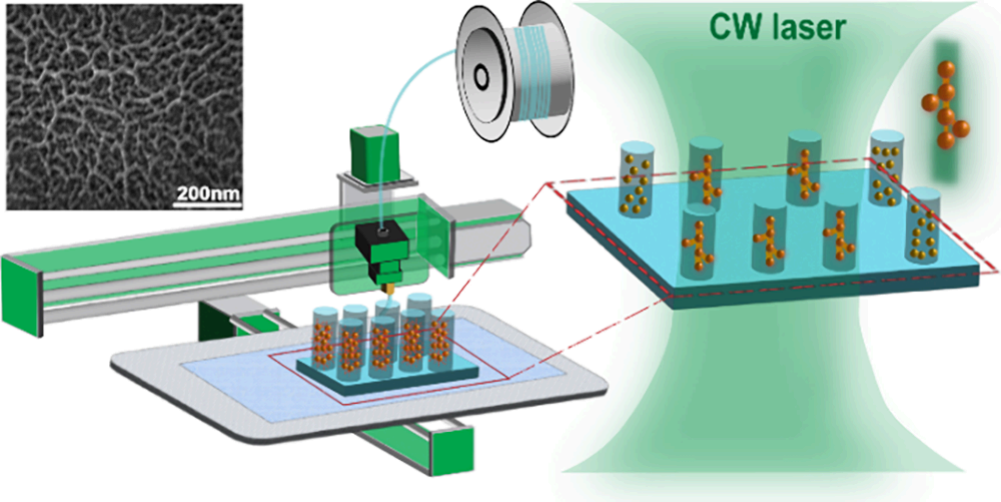

Additive manufacturing known as 3D printing has transformed the material landscape, with intricate structures and rapid prototyping for modern production. While nanoscale 3D printing has made significant progress, a critical challenge remains in the rapid, high-throughput tailoring of complex nanostructures. Here, we present a 3D printing-facilitated, light-driven assembly technology for rapid surface patterning consisting of complex particle nanonetworks with balanced fabrication resolution and processing scalability. This innovative approach seamlessly integrates top-down 3D printing (i.e., fused deposition modeling (FDM)) of digitally encoded patterns with bottom-up nanoparticle assembly (i.e., plasmonic light-driven techniques). The manufacturing-structure relationship of the generated nanonetworks within macroscale cylindrical patterning is investigated through programmatic modulation of critical processing parameters, including polymer rheology, chain-mode plasmonic resonances, nanoparticle dimensions, and peak optical intensity. The capacity of nanoscale 3D printing with optical adjustment can not only achieve high-resolution patterning but also offer precise control over large-scale geometries for applications in optical sensing.

## 1 Introduction

Tailoring nanoparticle assemblies enables precise control of material properties in advancing science and technologies.^1,2^ In particular, plasmonic nanoparticles demonstrate unique resonances attributable to the localized oscillation of electrons when interacting with light, known as surface plasmon resonances (SPR).^3^ Consequently, the ability to tailor plasmonic nanoparticle assemblies and hence resonant interactions underpin emergent functionalities such as subwavelength field enhancement, high surface sensitivity, electrical conductivity, and metamaterials,^4−7^ which promise tremendous potential in widespread applications in energy, environment, communication, computing, and particularly sensing.^8−11^ However, effectively incorporating solution-phase nanoparticle assemblies into deterministic large-scale solid architectures is challenging due to the intrinsic shapelessness of the liquid nanoparticle dispersion solution. To achieve integrated macroscopic shape control, conventional thin-film manufacturing techniques like casting, Langmuir–Blodgett, and doctor blading have been used to create film-shaped nanoparticle assemblies.^12−14^ Yet these techniques can only produce relatively simple external shapes or geometries, although the assembled nanoparticle patterns can be preserved.

In parallel, the development of 3D printing technologies, characterized by layer-by-layer additive manufacturing, garner unique capability of intricate and precise architecting, material prototyping, versatility, and sustainability.^15−17^ Widely adopted methods, such as fused deposition modeling (FDM), liquid deposition modeling (LDM), and digital light processing (DLP) have achieved great success. Featured by successively stacking two-dimensional patterns, these techniques can be used to construct complex three-dimensional structures with precise geometrical control.^15−18^ However, they generally have low printed resolutions (>100 μm) and lack versatile nanoscale patterning manufacturing capabilities and functionalities.^19−21^ Recently, emerging nanoscale 3D printing techniques, such as two-photon polymerization (2PP) and electrohydrodynamic (EHD) printing, have pushed feature sizes down to the nanometer regime (Typical 3D printing techniques for self-assembly of Au summarized in Table S1). However, these techniques still face challenges, including slow printing speeds and high costs, and often suffer from limited material choices and inefficient or complex processes.^22−24^ A critical technical barrier remains in developing an accessible high-resolution manufacturing route integrated with high-throughput 3D printing programmability that can lead to functional materials at the nanoscale.^25−27^

In this work, we uniquely integrated 3D printing with plasmonic light-driven nanoparticle self-assembly to construct nanoparticle patterns while retaining macroscale prototyping and geometrical control. Our approach utilizes the FDM 3D printing process by incorporating gold nanoparticles into the printing ink, such as Pluronic F-127 (Pf-127), to first define macroscopic architectures. We then actively guide the bottom-up assembly of the incorporated gold nanoparticles (AuNPs) by harnessing the photothermal properties, leveraging the SPR of Au NPs under light excitation at the specified wavelength. Specifically, when AuNPs are excited by light at their SPR frequency, the resonant oscillation of conduction band electrons facilitates the efficient absorption of incident radiation and its rapid conversion to heat through nonradiative relaxation and electron–lattice collisions.^28,29^ This highly localized photothermal effect generates steep thermal gradients in the vicinity of AuNPs, which induces thermophoretic forces^30^ surrounding nanoparticles along the optical path within the printing ink. By optically tuning the excitation light intensity, the distribution of these thermal fields can direct the nanoparticle diffusion and assembly in a controllable fashion. This synergistic integration of top-down 3D printing and optical-guided assembly enables novel hierarchical nanoto-macro structure constructions. By exploiting the plasmonic spectral changes of Au nanonetworks and the tunable nature of Pf-127, the sensor can simultaneously detect changes in environmental factors such as temperature, pH, analyte concentration, and even mechanical deformations, making it suitable for optical sensors in healthcare and robotics.

## 2 Experimental Section

### 2.1 Materials

Cetyltrimethylammonium bromide, hydrogen tetrachloroaurate(III) trihydrate (HAuCl_4_·3H_2_O), ascorbic acid, sodium citrate, and deionized water were used in this research. Pluronic f-127 ((C3H6O·C2H4O)_*x*_) with a molecular weight of 164.20 g/mol was purchased from Sigma-Aldrich (CAS #9003–11–6). Polylactic acid (PLA) 3D printer filament was purchased from Hatchbox. All materials were used as received.

### 2.2 Synthesis of AuNPs

Among various synthesis techniques, the in situ generation of AuNPs through the reduction of HAuCl_4_, resulting in citrate-stabilized AuNPs, has retained its pre-eminence since its inception by Turkevich in 1951.^31^ The Au seeds were synthesized using the Frens method.^32^ In a succinct overview, a solution comprising 100 mL of 2.5 × 10^–4^ M HAuCl_4_ (9.84 mg of HAuCl_4_ in 100 mL of water) was heated to 120 °C in an oil bath while being vigorously stirred for 30 min. Subsequently, 10 mL of a 1% sodium citrate solution was introduced into the heated solution and the boiling process continued. After 20 min, the solution underwent a discernible transformation from colorless to a ruby-red hue, indicative of the successful formation of AuNPs in the solution. To the resulting growth solution, composed of a mixture containing 2.5 × 10^–4^ M HAuCl_4_ and 0.01 M CTAB (0.886 mg of HAuCl_4_ + 32.800 mg of CTAB), 9 mL was added. After this, 50 μL of a freshly prepared 0.1 M (0.88 mg) ascorbic acid solution was gently stirred into the flask for 2 min. Finally, 0.5 mL of Au-seed solutions were introduced into the flasks. The mixture was then incubated at 30 °C in a water bath for 6 h.

### 2.3 3D Printing and Ink Formulation

The substrate, measuring 50 mm in length, 1 mm in thickness, and 30 mm in width, was fabricated through 3D printing using a Flashforge Creator 3 Pro printer. The substrate features cylinders with a diameter ranging from 1 to 2 mm, spaced at intervals of 5 mm. PLA filament was employed for the 3D printing process. In the ink formulation, Pf-127 was gradually dissolved in distilled water at concentrations of 22.5, 25, 30, and 35 wt %. These solutions were stored at 4 °C, below the transition temperature, to maintain the colloidal Au colloidal state. Subsequently, AuNPs were introduced into different weight percentages of Pf-127.

### 2.4 Laser Setup and Discussion

Employing a LambdaPro laser operating at 532 nm continuous wave (CW) and producing 1 W of power, we intricately managed the assembly of AuNPs. To regulate the laser’s intensity, we utilized a Continuously Variable Neutral Density (ND) Filter (NDC-50C-4M) with a 50 mm diameter, spanning an optical density range from 0.04 to 4.0, through an AC254-100-A-f = 100 mm, Ø1″ lens. With laser focus, it usually takes the Gaussian distribution,^33,34^ and the beam spot size and waist spreading out light intensity can be described by
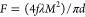
1
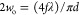
2Where *F* is the spot size, *w*_o_ is the beam waist, *d* is the beam diameter at the lens, *M*^2^ is the beam’s quality factor, λ is the wavelength, and *f* is the focal length. The measured *F* and *w*_o_ from the above equations are 0.027 and 0.0135 mm, respectively.

### 2.5 Characterizations

#### Scanning Electron Microscopy (SEM)

The morphology (i.e., shape, size, and size distributions) of the AuNPs, and cross-section of polymer-embedded AuNPs were analyzed by SEM (Focused Ion Beam from Auriga (Zeiss)) with a voltage ranging from 5 to 20 kV. Nanoparticle samples were deposited on a silicon substrate, and the polymer samples were coated with gold (Au) for better conductivity with a coating duration of 2 min. In our investigation, we utilize the plasmon-induced photothermal effect to macroscopically assemble the nanonetwork. It is essential to note that the dicing process could potentially influence the morphology and structure of our original sample. Moreover, our resin constitutes a nonconductive Pf-127, thus susceptible to damage from the high-voltage electron beam used in Transmission Electron Microscopy (TEM). This poses a risk of inaccurately reflecting the assembly morphology of the sample, so SEM is preferred over TEM for the imaging.

#### UV–Vis Spectroscopy

The Lambda 950 UV/vis/NIR is a double beam, double monochromator, all-reflecting optical system that operates in the ultraviolet (UV), visible (Vis), and near-infrared (NIR) spectral range: deuterium and tungsten halogen light sources. The usable wavelength range is 200 to 2500 nm. The presence of AuNPs in the polymer was confirmed by UV–vis analysis.

#### Optical Microscopy

The Zeiss AXIO Observer.D1 was used to capture dark-field images of the AuNPs. Additionally, an analysis of transmission percentage was conducted, facilitating the calculation of absorption.

#### Rheology Behavior

The rheology behavior of Pf-127 was measured via a rheometer (Discover Hybrid Rheometer HR2, TA Instruments). A parallel plate geometry was applied for the viscosity measurement, where the shear rate varied from 0.001 to 8000 s^–1^. The storage and loss moduli were obtained by performing amplitude sweeps with varying stress from 0.02 to 1000 Pa at an angular frequency of 1 rad s^–1^.

#### Thermal Stability

The thermal stability of the Pf-127 and 25.0Pf-127/Au was evaluated by a Thermogravimetric Analyzer (TGA) (Discovery TGA 550, TA Instruments), measuring the mass change in polymer and nanocomposites caused by thermal transition (e.g., moisture evaporation, degradation) process. TGA results in the nitrogen environment showed the sample’s thermal transitions with a heating rate of 10 °C min^–1^ from room temperature (RT) (i.e., 24 °C) to 620 °C. The curing kinetics of Pf-127 were analyzed using Differential Scanning Calorimetry (DSC) (Discovery DSC 250, TA Instruments). All operations were performed under a nitrogen atmosphere with a sample weight of ∼8 mg. All samples were first heated from RT (i.e., 25 °C) to 75 °C at a 10 °C min^–1^ temperature ramp. Then, the samples were cooled to −20 °C at a ramp rate of 10 °C min^–1^, respectively.

## 3 Results and Discussion

### 3.1 Light-Driven Nanoscale Assembly via 3D Printing

**Figure 1:**
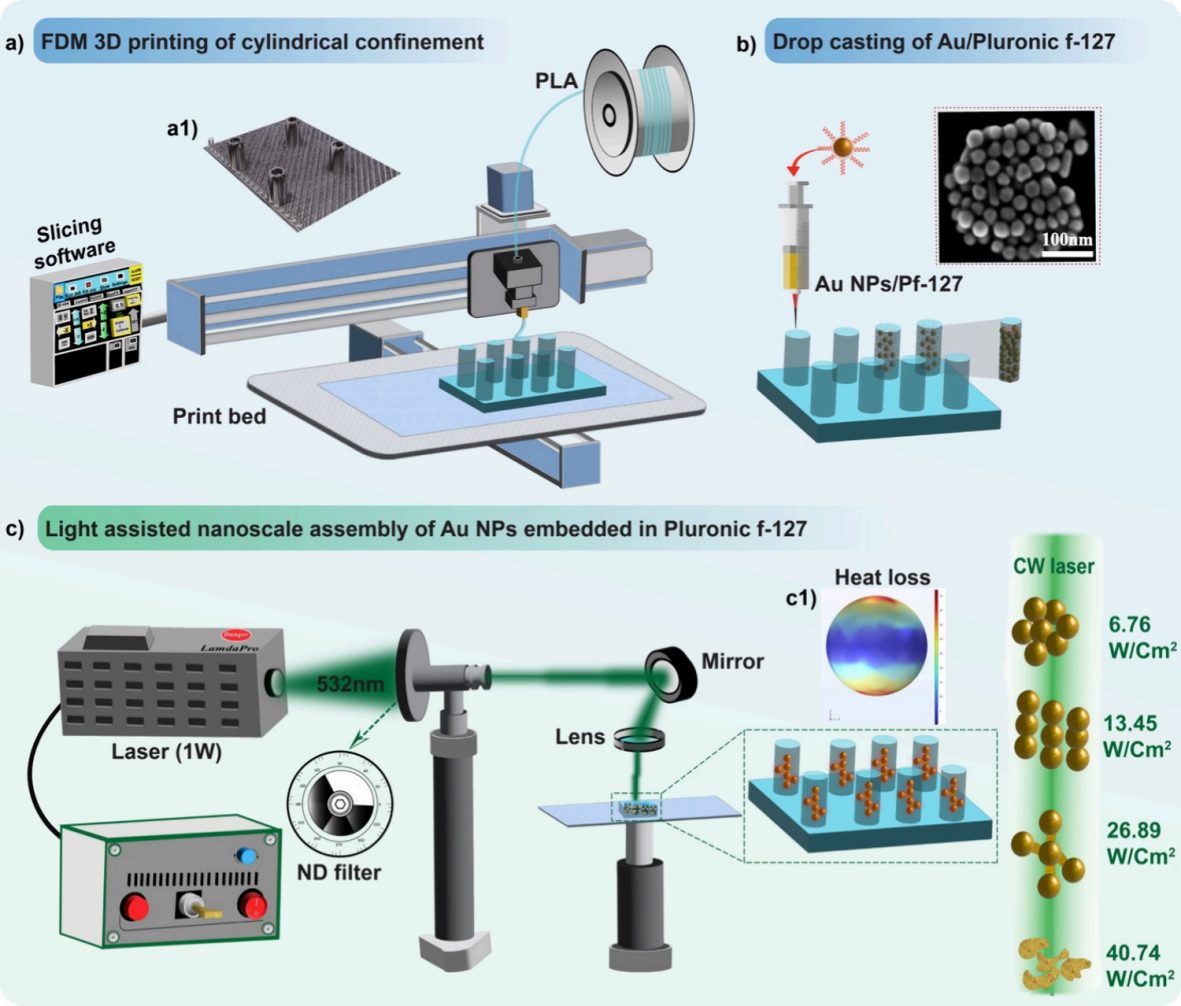
Schematic illustration of the hybrid 3D printing process: (a) creation of the designed cylindrical structures using FDM, (a1) visual representation of the 3D printed cylindrical formations, (b) ink deposition of Pf-127/AuNPs mixture into the cylinders enclosed with the SEM of AuNPs, (c) laser setup of the nanoscale assembly AuNPs within the confines, and (c1) the 3D simulation spectrum of light-induced heat generation.

Our light-driven 3D printing assembly technique takes advantage of the easy accessibility of induced nanoparticle assembly and rapid FDM printing prototyping. As a demonstration, we use Pf-127/AuNPs ink, which features great solubility, ease of cure, and strong SPR from AuNP.^35−39^Figure 1 illustrates our methodology for the nanoscale assembly of AuNPs through a fabricated printing process. Patterned surface templates, essential for subsequent nanoparticle assembly, were 3D printed using FDM. PLA filaments underwent FDM printing at temperatures approaching the melting point (≈ 205 °C). The heated filaments were extruded through a nozzle onto the printing platform, as depicted in Figure 1a, allowing precise control of surface topology for later confined particle nanostructures through layer-by-layer deposition. The melted PLA solidified promptly upon deposition during FDM printing, facilitated by the temperature differential between the extrusion nozzle and the substrate. A cylindrical geometry was designed, which can lead to higher effective AuNP concentrations and reduce the diffusion distances between particles due to the limited space, facilitating the demonstration. Initially, a computer-aided design (CAD) model was crafted, outlining a flat substrate with embedded cylinders for the subsequent deposition of the Pf-127/AuNPs ink. The surface feature was printed along the longer edge of the substrate to dictate the polymer layer orientation. The FDM 3D printing process fabricated the template layer-by-layer, generating a surface template featuring cylindrical patterns of varying diameters (e.g., tunable volume or area for the confined regimes) as seen in Figure 1a1.

Note that FDM and droplet-deposition-based Direct Ink Writing (DIW) can be combined as a hybrid 3D printing technique to leverage the strengths of both methods. FDM works by melting and extruding thermoplastic filaments layer by layer to create a 3D object, while DIW dispenses a continuous filament of a viscous ink or paste through a nozzle, allowing for the creation of complex structures with a wide variety of materials, as depicted in Figure 1b. Employing capillary action and microfluidic forces, nanoparticles are transported, confined, and oriented within microchannels, facilitating the assembly of AuNPs.^40^ Subsequently, these printed cylinders serve as material feeding sites for nanoscale nanoparticle assembly by light, as illustrated in Figure 1c. This plasmon light strategy involves subjecting the nanoparticles to light at the resonant SPR wavelength. Starting with individually separated nanoparticles and exploiting the power-dependent plasmonic excitations, we were able to link the AuNPs into chains and networks connecting nanoparticles into continuous assemblies across the cylindrical channels on a large scale. As seen in Figure 1c1, the SPR of AuNPs can efficiently generate a well-defined heat distribution surrounding the AuNP due to the photothermal effect, which can lead to directional AuNP self-assembly due to the plasmon-induced thermophoretic forces.

### 3.2 Au Synthesis and Characteristics

**Figure 2:**
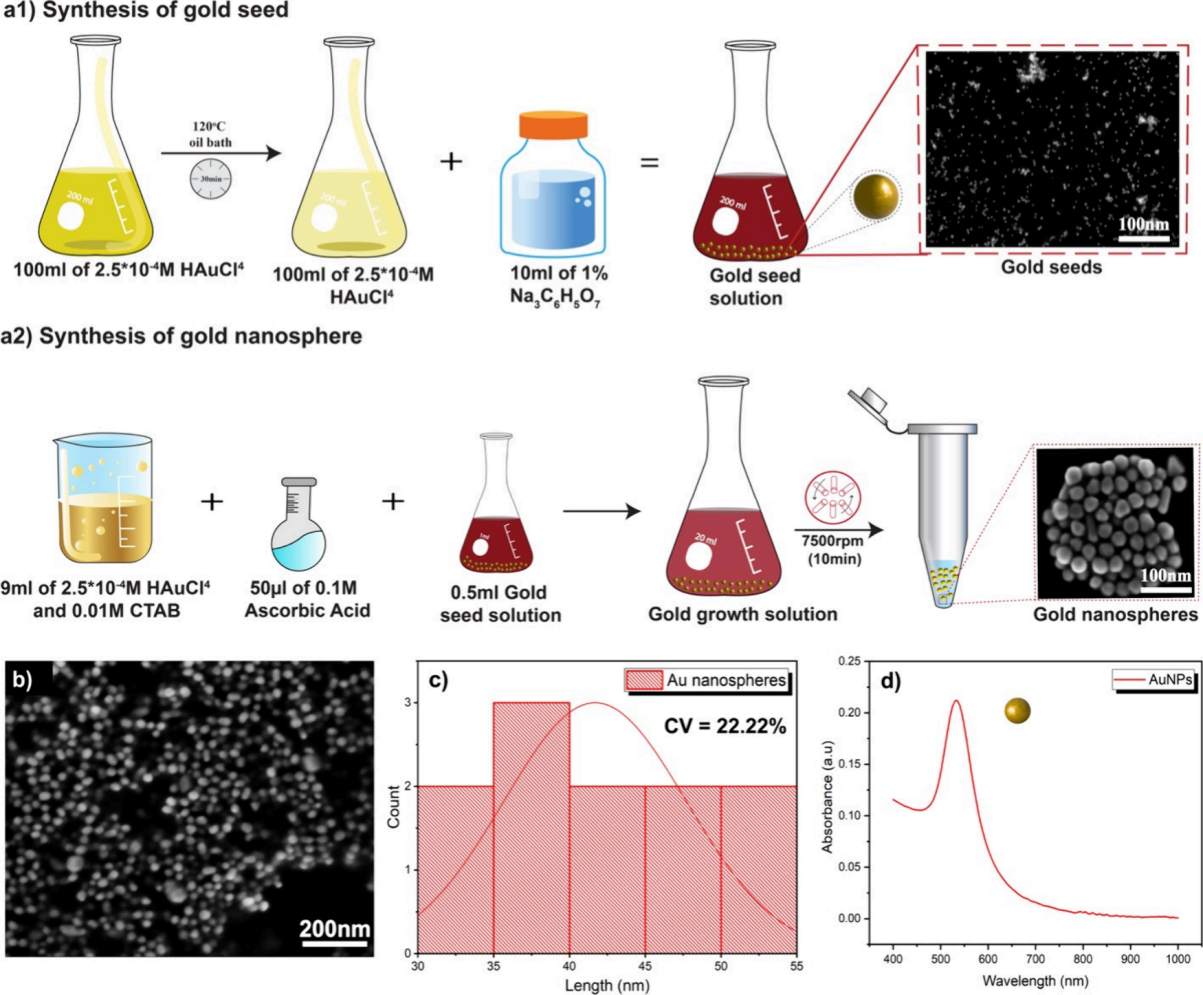
Illustrative depiction of the process stages including (a1) Au seed creation, (a2) Au growth solution preparation, (b) SEM images of Au nanospheres highlighting their morphology, (c) particle size distribution of Au nanoparticles with coefficient of variation (CV), and (d) optical absorption spectra of AuNPs solution.

To start with, we employed the seed-mediated growth method to fabricate AuNPs,^32^ following the detailed procedures outlined in the Experimental Section and Figure 2a1 and a2. Briefly, the HAuCl_4_ solution undergoes a process of boiling, followed by the prompt addition of trisodium citrate dihydrate under vigorous stirring. Subsequently, within a brief duration, a distinctive wine-red colloidal suspension is achieved, showcasing AuNPs seed with a discernible size of ∼18 nm (depicted in Figure 2a1. CTAB and ascorbic acid were then added to Au seed solution and kept at 30 °C for 6 h.

The SEM image of the resulting AuNPs indicates that the nanoparticles predominantly adopt a spherical shape (as depicted in Figure 2b). The quantitative analysis by employing ImageJ reveals the average diameter of the individual AuNPs was 45 ± 10 nm, with size distribution illustrated in Figure 2c. The measured optical absorption spectra of AuNPs are shown in Figure 2d. The only prominent peak observed at 532 nm in the UV–vis spectrum corresponds to the SPR of AuNPs, indicating the monodispersed AuNPs from synthesis.

### 3.3 Rheological and Curing Characteristics of Au/Pf-127 Inks

For 3D printing integration, ensuring a consistent distribution quality with uniform nanoparticle dispersion and regulated rheological characteristics is essential for the even deposition of AuNPs. Various wt % of Pf-127, when dissolved in water, are combined with different wt % of AuNPs in water, resulting in diverse concentrations as detailed in Table 1. This process is crucial for achieving stability and homogeneity in the dispersion of AuNPs, which is a pivotal factor in the subsequent deposition process.

**Table 1:** Different Compositions of Polymer Matrix and Nanoparticle Reinforcement^a^

	**Composition (wt %) in solutions**				**Au in solid samples**			
**Samples**	**Pf-127/Solvent(wt %)**	**Au (mg)**	**Water (mL)**	**Au/Water(wt %)**	**Au/Pf-127(wt %)**	***T***_**g**_**(°C)**	***T***_**mi**_**(°C)**	***T***_**c**_**(°C)**
Pf-127 Powder	100	N/A	N/A	N/A	N/A	N/A	N/A	35.5
22.5Pf-127/Au	22.5	9.84	1	0.984	4.37	–39.4	–14.5	12.0
25.0Pf-127/Au	25.0				3.93	–40.5	–16.4	10.7
30.0Pf-127/Au	30.0				3.28	–39.7	–14.8	5.3
35.0Pf-127/Au	35.0				2.81	–40.3	–13.6	0.5

a Note: *T*_m_ = 59.7 °C is the melting of Pf-127 powder. *T*_mi_ is the micellization temperature. *T*_g_ is the glass transition point. *T*_c_ is the crystallization point.

**Figure 3:**
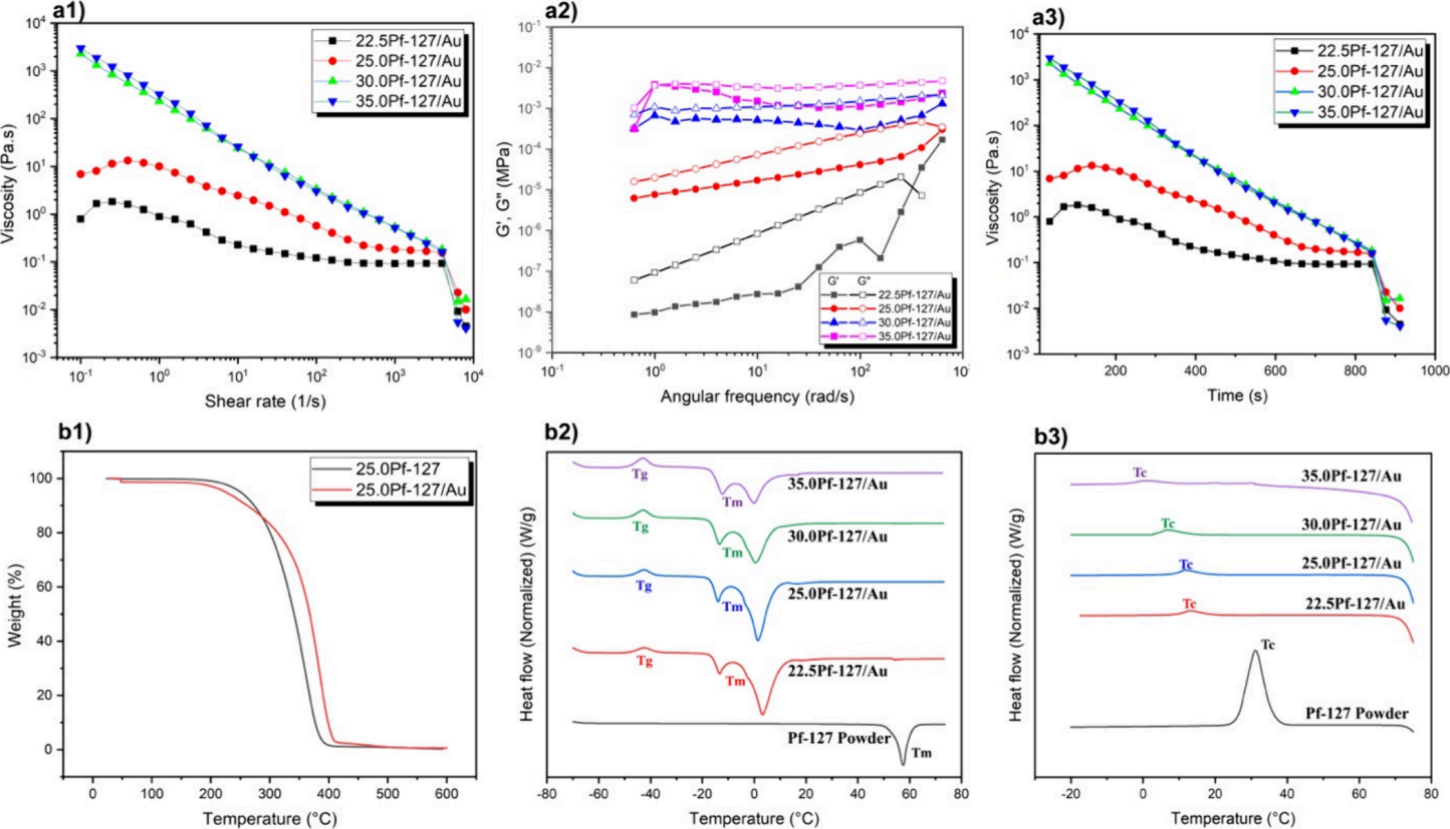
Exploration of the viscoelastic and solidification behavior of PF-127/AuNP mixtures: (a1) depicts the relationship between viscosity and shear rates ranging from 0.1 to 8000 s^–1^; (a2) shows the comparison of storage modulus (*G*′) and loss modulus (*G*″) against angular frequency; (a3) illustrates the time-dependent viscosity changes; (b1) presents thermogravimetric analysis (TGA) of PF-127 with and without Au, detailing weight percentage against temperature (°C); and (b2, b3) display differential scanning calorimetry (DSC) results, exhibiting the heat flow to temperature (°C).

In addition to achieving uniform dispersion, the rheological properties of the inks play a critical role in ensuring the successful deposition and patterning of nanoparticles. Figure 3a1 illustrates the rheological behavior of various polymer weight concentrations while keeping the AuNP’s concentration constant. The viscosity increases with higher concentrations of polymer wt %. When exposed to room temperature, 25 wt % of these concentrations showed excellent stability and perfect solidification time required for the assembly of AuNPs using light, In contrast, 30 and 35 wt % solidified more quickly than 25 wt % due to an increase of polymer chains, facilitating faster micellization and gelation.^41,42^ At low temperatures (e.g., below 10 °C), Pf-127 exists as unimers (single polymer chains) with a hydrodynamic diameter (dH) of around 9 nm. As the temperature increases to around 25 °C, the PEO (poly(ethylene oxide)) blocks of Pf-127 become dehydrated, leading to the formation of micelles with a dH of approximately 30 nm.^43^ This micellization is driven by the thermoresponsive behavior of the PEO units. Further increasing the temperature to around 30 °C triggers the sol–gel transition, where the Pf-127 solution transforms into a liquid crystal gel state. This transition occurs due to the increased interactions between the dehydrated PEO blocks and the hydrophobic PPO (poly(propylene oxide)) blocks within the micelles. So higher concentrations such as 30 and 35 wt % have more polymer chains to undergo micellization and subsequent gelation.^41,42,44^ Besides, we also calculated the solidification time of different wt % of Pf-127 solution, and 25 wt % solidifies in ∼2 min, while 30 and 35 wt % solidify within 30 s upon exposure to RT. Even, at lower concentrations, water molecules bonded to Pf-127 through hydrogen bonding. However, an increase in the amount of Pf-127 led to aggregation, forming a 3D network via hydrogen bonding, thereby reducing the fluidity of the dispersion. Viscoelastic properties were explored through an oscillation sweep at a constant strain rate, recording storage modulus (*G*′) and loss modulus (*G*″) as a function of frequency (rad/s) at fixed stress (0.015 Pa), as shown in Figure 3a2. The inks demonstrated a transition from a liquid to a solid-like nature around frequencies of ≈350 rad/s for 22.5 wt % Pf-127, with frequency increasing as the polymer concentration increased. The dominance of *G*″ over *G*′ indicates that the Pf-127/water/AuNPs dispersion is suitable for high shear-rate processing methods using solution-based deposition approaches. Figure 3a3 shows the recovery time of the ink: after being sheared in the nozzle, the Pf-127/water/AuNPs ink has to recover a relatively high viscosity in a limited amount of time to freeze the assembly of AuNPs. It shows the time evolution of the viscosity of different concentrations of Pf-127/AuNP after applying a step shear rate, which confirms that the presence of AuNPs does not affect the gel recovery time.

Figure 3b1 illustrates the TGA curve for both Pf-127 and the nanocomposite. The exceptional thermal conductivity of AuNPs contributes significantly to enhancing the stability of Pf-127, making it more resilient to thermal degradation.^45^ Moreover, AuNPs play several important roles in this enhancement: they restrict the mobility and segmental motion of the polymer chains,^45^ serve as a physical barrier,^46^ and catalyze the formation of a protective char layer on the polymer surface during thermal decomposition.^47^ These actions collectively impede the degradation process, increasing from 391 to 408 °C, beginning at a concentration of 3.9 wt %, and decreasing to 0.4 wt % by 600 °C.

To comprehend the phase transitions occurring within the materials, DSC curves were recorded for both different wt % of Pf-127/Au (as depicted in Figure 3b2 and b3. The initial event in heating a Pf-127 water solution is known as micellization, where individual polymer chains, termed “unimers”, aggregate to form micelles. These micelles feature a central core composed of hydrophobic PPO segments and an external corona comprised of hydrophilic PEO segments.^48−50^ Micellization is an endothermic process occurring around −13.6 to −16.4 °C depending on the concentration of Pf-127 in water. The first endothermic peak corresponds to the micellization process with an onset at the critical micellization temperature. The second peak is associated with gelation, occurring at the critical gelation temperature. The micellization process leads to the formation of micelles, transitioning the solution from a low-viscosity liquid state to a gel state (as illustrated in Figure 3b2). After micellization, the gelation phenomenon transpires at elevated temperatures ranging from 0.5 to 35.5 °C and is a crucial step in the development of a structured gel from Pf-127 solutions (as seen in Figure 3b**3**). This phenomenon encompasses both the growth and aggregation of micelles. The rise in the concentration of Pf-127 wt % enhances the stability and size of these micellar structures, necessitating higher temperatures for their disruption. Unlike *T*_m_ and *T*_c_, *T*_g_ is typically influenced more by the polymer’s molecular structure than its concentration, so *T*_g_ remains constant at around −40 °C. The curing time for 25 wt % Pf-127 solution took ∼1 min when exposed to room temperature (RT) and the curing time decreased when the concentration of the polymer increased.

### 3.4 Structural Characteristics

**Figure 4:**
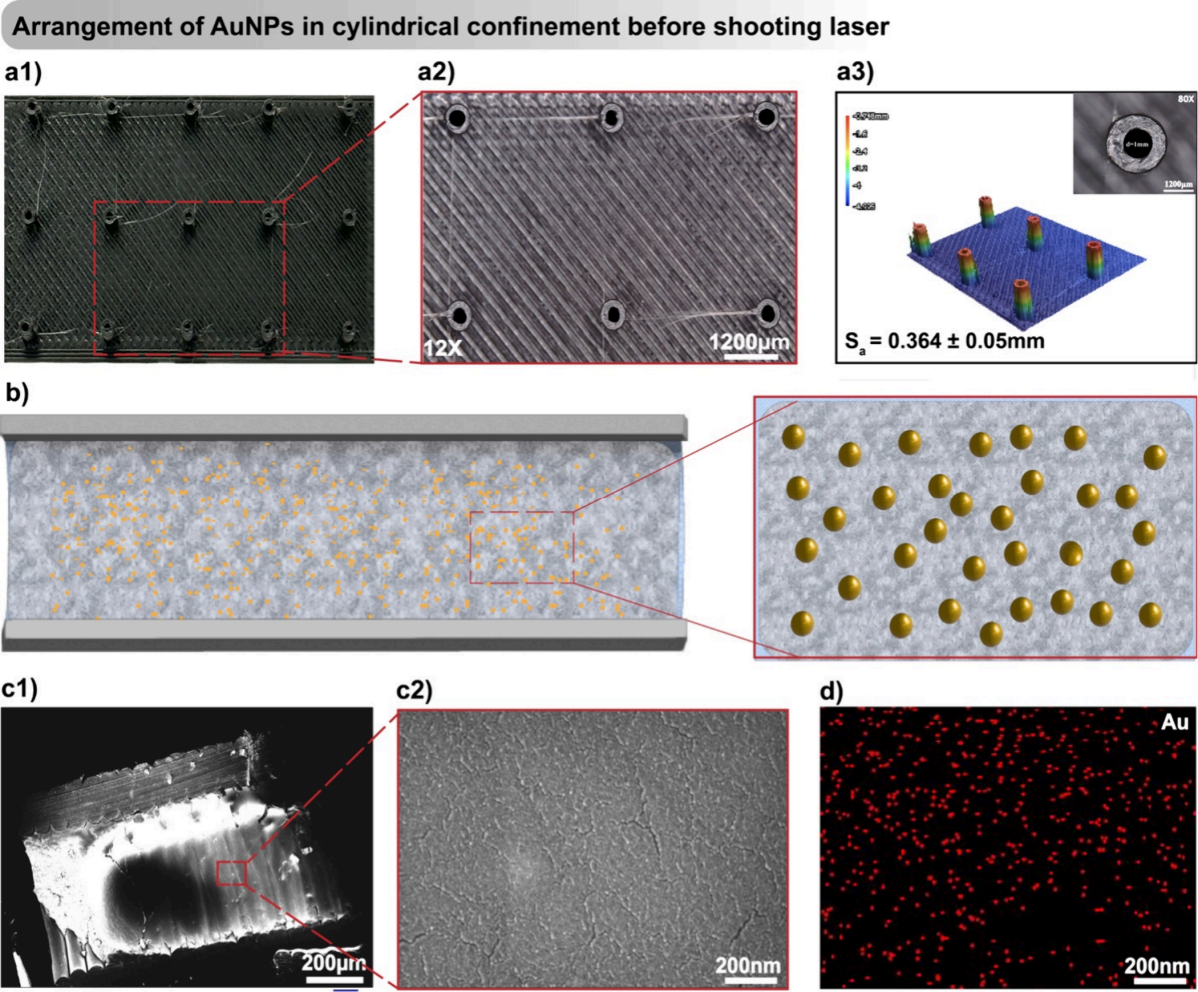
Visualization and analysis of the surface and structural features of 3D printed cylindrical patterns are provided through (a1, a2) optical images displaying the patterned substrate from a top view, and (a3) 3D optical profilometry detailing surface roughness and cylinder details. The spatial arrangement of Au NPs within cylindrical confines and the characterization of the 3D printed substrate are shown in (b), which schematically represents the AuNP organization before laser treatment. Cross-sectional details of the substrate are captured in an SEM image (c1), with a magnified view (c2) revealing the random arrangement of particles, while energy-dispersive X-ray spectroscopy (EDS) mappings (d) depict the spatial presence of gold (Au) within the substrate.

As outlined in Figure 1, the PLA filaments underwent a 3D printing process employing FDM technology. The 3D printing parameters, meticulously outlined in Table 2, were utilized to fabricate cylinders with varying diameters. Each cylinder was systematically printed at a fixed distance of 10 mm from each other, featuring diameters of 1, 1.5, and 2 mm while maintaining a consistent height of 4 mm (as seen in Figure S1). The resulting 3D-printed cylindrical pattern of diameter 1 mm is visually depicted in Figure 4a1, complemented by zoomed-in optical images for closer examination (as illustrated in Figure 4a2). The choice of a cylinder with a smaller diameter, specifically 1 mm, resulted in structures with a notably smooth surface finish. These structures exhibited a quantified surface roughness (*S*_a_) of approximately 0.364 mm. This superior smoothness can be attributed to the smaller inner diameter of the cylinder, a phenomenon evident in the detailed 3D imaging provided in Figure 4a3. Conversely, larger diameters (i.e., 1.5 and 2 mm) showcased an increase in Sa, measuring approximately 0.594 and 0.759 mm, respectively, as evidenced in 3D images (as depicted in Figure S5). The optical imaging presented in Figure 4a3 further elucidates the inner diameters of the cylinder.

**Table 2:** Printing Parameters of the 3D Printed Substrate

**Polymer**	**Printing temperature (°C)**	**Bed temperature (°C)**	**Printing speed (mm/s)**	**Nozzle diameter (mm)**	**Infill (%)**	**Infill pattern**	**Layer height (mm)**
PLA	205	45	60	0.4	15	Line	0.2

In the investigation of the AuNPs assembly, the process involves depositing a colloidal solution of Pf-127/Au onto a 3D-printed cylindrical pattern through drop casting, as shown in Figure 4b. This method employs a nuanced interplay of short-range forces, such as van der Waals, capillary, and long-range forces, specifically gravity, during the sequential stages of solvent evaporation and polymer curing.^27,51^ The convective flow, driven by capillary action, confines the droplet within the microchannels. As the nanoparticles experience the solvent–air interface, they orient with their primary axis parallel to the contact line, subjected to a downward gravitational force. Upon stabilization of the suspension within the microchannel, gravitational forces further contribute to the sedimentation of AuNPs.^52^ This results in a layer-by-layer deposition of particles at the bottom of the channels following the evaporation of water. Without light-guided assembly, the random and noncontinuous arrangement of AuNPs in Pf-127 ink is observed (Figure 4**(c1-c2)**), confirmed by EDS Au element mapping analysis (Figure 4d andFigure S2).

### 3.5 Optical Field-Assisted Self-Assembly

**Figure 5:**
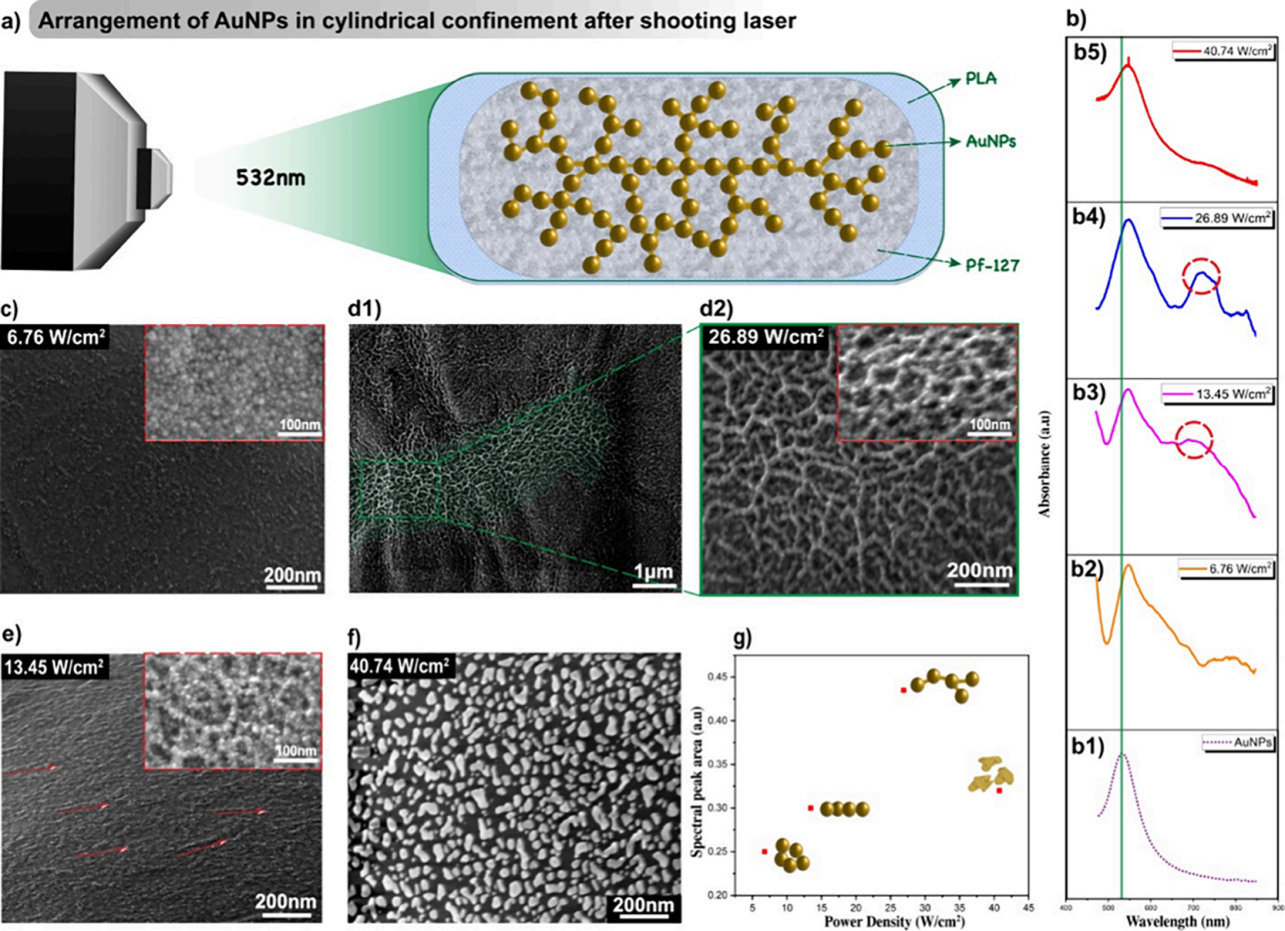
Evolution of AuNPs in cylindrical confinement. (a) Schematic representation of Au NP arrangement following laser exposure, (b1–b5) optical absorption spectra of AuNPs solution and Pf-127/AuNPs in cylindrical confinement exposed to different laser power densities, SEM image revealing the (c) random orientation of AuNPs when exposed to 6.76 W/cm^2^, (d1) cross-sectional view of the 3D printed substrate with (d2) magnified view revealing a distinctive chain-like pattern formed by the AuNPs at power 26.89 W/cm^2^ due to increased thermophoretic force, (e) more line-like threading of AuNPs when exposed to 13.45 W/cm^2^ due to SPR excitation, (f) shape change of AuNPs when exposed to 40.74 W/cm^2^ due to the plasmonic photothermal effect, and (g) spectral peak area of 700 to 850 nm vs power density.

To assemble the AuNPs with printing, we first determine the plasmonic resonance via UV–vis measurement showing the prominent plasmon resonance of the synthesized AuNPs at around 532 nm (Figure 5b1). Therefore, a 532 nm laser was employed to induce the AuNPs assembly (Figure 5a) and EDS spectrum analysis confirms the elemental composition of Au after the photothermal effect (Figure S3). At this wavelength, the light energy can efficiently trigger the resonant oscillation of SPR. As the electrons in AuNPs oscillate at this SPR frequency, the light’s energy gets absorbed locally and is converted into heat through several processes. First, collisions between resonating electrons and the metallic lattice structure generate thermal energy. Second, the nonradiative relaxation of excited electrons back to the ground state releases absorbed light energy as heat. This plasmonic photothermal process under light excitation concentrates heat generation close to the AuNP surface, generating a temperature gradient around the illumination beam spot area, and hence thermophoretic force toward the beam center.^53^ Therefore, the AuNPs will tend to diffuse, align, and assemble along the beam path. Interestingly, we found that the patterns of AuNP assembly are highly dependent on the excitation light powers (laser parameters are outlined in Table 3). The constant time of 10 s has been maintained throughout the study because when the exposure time is increased beyond 30 s, the substrate gets damaged as seen in Figure S4. To clarify the impact of gold spraying treatment during SEM imaging, we also performed the EDS Au elemental spectrum using carbon coating of the sample. It can be observed that Au displays a major peak of Mα 2.13 keV from gold nanoparticles and peaks of carbon and oxygen mainly come from polymer, as seen in Figure S5.

**Table 3:** Laser Parameters for the Assembly of Gold Nanonetworks

**Power**	**ND filter (deg)**	**Beam diameter (mm)**	**Wavelength (λ)**	**Focal length (mm)**	**Power Density (W/cm**^**2**^**)**	**Focal spot size (mm)**	**Depth of field (mm)**	**Time (s)**
1 W	0	2.5	532	100	40.74	0.027	2.17	10
	120				26.89			
	240				13.45			
	300				6.76			

Initially, at a low power density of 6.76 W/cm^2^, the AuNPs network remains a random configuration (Figure 5c), but the assembly initiation can be observed with broadened line width of the SPR resonance peak (Figure 5b2). It also shows a slight redshift to the maximum peak position at 540 nm, this is the index change of the polymer due to the local heat.^54^ As increasing power density to 13.45 W/cm^2^, the distinctive line-like threading of AuNPs was observed (Figure 5e). Under this condition, the SPR excitation triggers a larger localized heat gradient, compelling the AuNPs to move toward the beam path. But at this power, the thermophoretic force from photothermal heating is still not large enough to pull all AuNPs in the vicinity toward the center of the beam path, AuNPs threading is observed occasionally. As the power further increases (26.89 W/cm^2^), the thermophoretic force increases such that a great number of AuNPs were pulled toward the center of the laser beam (see the thermophoretic force discussion in the Supporting Information). Due to the Gaussian beam shape (see the laser setup and discussion in the Experimental Section), the laser power decays from beam center to edge and hence the decay of the local photothermal gradient. The AuNPs assembly tendency gradually reduces from beam center to edge. Thus, nanoparticle networking along the beam path has been observed (Figure 5d1 and d2). As a result, the pronounced peaks (720 and 840 nm) at longer wavelengths were shown in the absorption spectra as rising incident power from 13.45 W/cm^2^ to 26.89 W/cm^2^ (Figures 5b3 and b4). These emerging peaks are typically indicative of the formation of nanoparticle threads or networks.^55^ The collective optical properties arising directly from the AuNPs assemblies can be used in sensors, actuators, and surface-enhanced Raman scattering (SERS).^2,56^

Upon further increasing the power density to 40.74 W/cm^2^, a dramatic shape change of AuNPs was observed as the heightened heat generation from the plasmonic photothermal effect induces fusion and damage to the AuNPs (seen in Figure 5f). In this case, both absorption peaks of network assemblies and the initial SPR of AuNPs have been suppressed (Figure 5b5) due to the observed nonassembled discrete nanoparticles. In this case, the laser energy tends to fuse AuNPs instead of the assembly. The overall assembly stages are depicted as a function of laser excitation power (Figure 5g). The spectral peak region from 700 to 850 nm has been integrated using baseline corrections to characterize the aggregation states of AuNPs at various powers. While increasing the power intensity is increased, the peak area increases as nanoparticles assemble into networks and aggregate. Further increasing the power will lead to high enough local heat to fuse the AuNPs such that the peak area is due to decreased aggregation. The power-dependent understanding and investigations provide us with the guideline for morphology control of nanoparticles and nanonetworks within a 3D printing assembly.

## 4 Conclusion

In summary, we have demonstrated a nanoscale 3D printing-assembly technology that addresses the traditional additive manufacturing constraints by offering high resolution and high throughput manufacturing capabilities. By developing the unique printing ink of Pf-127/AuNPs, we fabricated numerous nanoscale nanoparticle assembly configurations, including threads, networking, and AuNPs shaping under light excitation as a function of power density. The establishment of nanonetworks is intricately dependent upon the delicate balance between the photothermal gradient, efficiency, and nanoparticle photothermal stability. Our findings hold great potential in optical sensing applications for the attainability of precise nanoscale control with a 3D printing customization through light manipulation.

## Supporting Information Available

Optical images and printing parameters of the 3D printed substrate; theoretical prediction of AuNPs in polymer; EDS analysis before and after shooting laser; deformation of the 3D printed substrate; mechanism behind the formation of nanonetworks and the respective laser parameters (PDF)
