# Supplementary material for: Light-Driven Nanonetwork Assembly of Gold Nanoparticles via 3D Printing for Optical Sensors
Source: ACS Appl Nano Mater. 2024 May 23;7(24):27998–8007. doi: 10.1021/acsanm.4c01673 (PMC11686463; doi:10.1021/acsanm.4c01673)
Supplement: Supplementary file 1 — an4c01673_si_001.pdf [file an4c01673_si_001.pdf]

Supporting Information

Light-Driven Nanonetwork Assembly of Gold Nanoparticles via 3D Printing for Optical Sensors

*Arunachalam Ramanathan[a,^], Shuai Feng[b,^], Abhishek Saji Kumar[b], Sri Vaishnavi Thummalapalli[a], Martin Taylor Sobczak[a], Lindsay R. Bick[a], Kenan Song[c,d *], Sui Yang[e, *]*

[a] Mechanical Engineering, College of Engineering, University of Georgia, 302 E. Campus Rd, Athens, 30602, USA

[b] Materials Science and Engineering, School for Engineering of Matter, Transport and Energy (SEMTE), Ira A. Fulton Schools of Engineering, Arizona State University (ASU), Tempe, AZ 85287, USA

[c] Associate Professor of Mechanical Engineering, School of Environmental, Civil, Agricultural, and Mechanical Engineering (ECAM), College of Engineering, University of Georgia, Athens, GA 30602, USA

[d] Adjunct Professor, The School of Manufacturing Systems and Networks (MSN), Ira Fulton School of Engineering, Arizona State University, Mesa, AZ 85212, USA

[e] Assistant Professor of Material Science and Engineering, School for Engineering of Matter, Transport and Energy (SEMTE), Ira A. Fulton Schools of Engineering, Arizona State University (ASU), Tempe, AZ 85287, USA

^ co-first-author

*Corresponding author, Email: kenan.song@uga.edu; sui.yang@asu.edu

Table of Contents

1. 3D Printed Substrates....................3

2. EDS Analysis Before Shooting Laser....................3

3. EDS Analysis After Shooting Laser....................4

4. Deformed 3D Printed Substrate when exposed to laser....................5

5. EDS Analysis in the presence of carbon coating....................5

6. Thermophoretic Force and Discussion....................6

7. Literature survey on self-assembly of AuNPs via different 3D Printing Techniques 7

7. References....................8

Table of Figures

Figure S1. 3D optical profilometry images demonstrating surface roughness, thickness and zoomed-in optical image of the cylinder of various diameters....................3

Figure S2. EDS images illustrating the spatial distribution of (Carbon (C), Oxygen (O), and Gold (Au) elements before the laser....................4

Figure S3. EDS spectrum depicting the spatial distribution of (Carbon (C), Oxygen (O), and Gold (Au) elements after the laser....................4

Figure S4. Laser effect on 3D printed substrate when the power density 40.74 W/cm^2 exposed for 30 seconds....................5

Figure S5. EDS images illustrating the spatial distribution of (Carbon (C), Oxygen (O), and Gold (Au) elements in the presence of carbon coating....................5

Table of Tables

Table S1. Literature reviews on AuNPs assembly via different 3D printing techniques 7

1. 3D Printed Substrates

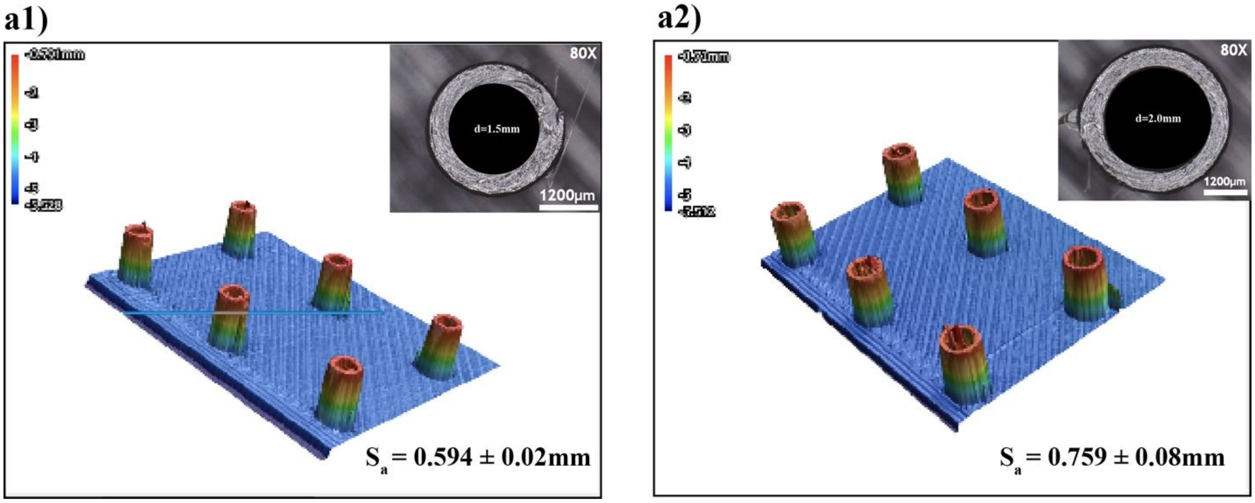

Figure S1. 3D optical profilometry images demonstrating surface roughness, thickness and zoomed-in optical image of the cylinder of various diameters.

2. EDS Analysis Before Shooting Laser

The elemental composition is characterized by a predominant percentage of carbon, followed by oxygen and gold. The heightened carbon content is attributed to Pf-127, a polymer with a substantial carbon component derived from its ethylene oxide and propylene oxide units. The oxygen peak corresponds to oxygen atoms present in both Pf-127 and other organic components. The detection of gold in the spectrum confirms the successful incorporation of AuNPs within the Pf-127 matrix. The Au signal is lower in percentage compared to carbon and oxygen as it signifies the low loading presence and uniform dispersion of AuNPs in the composite material.

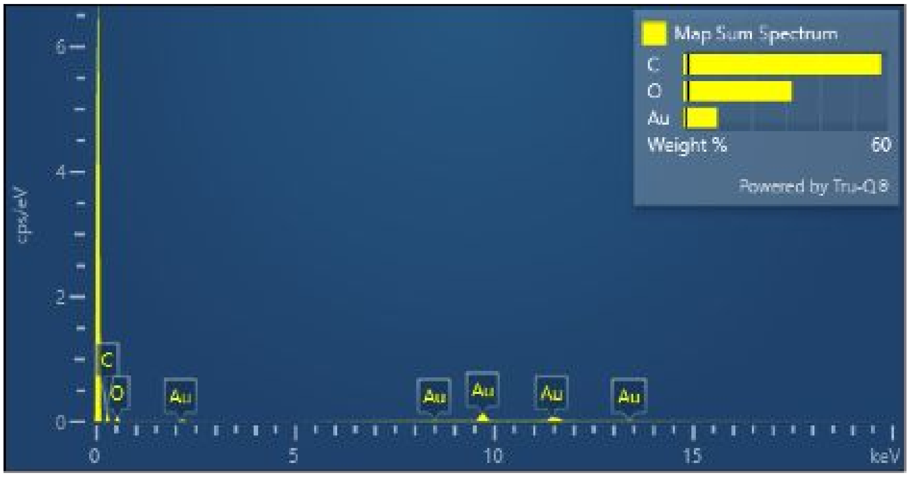

Figure S2. EDS images illustrating the spatial distribution of (Carbon (C), Oxygen (O), and Gold (Au) elements before the laser.

3. EDS Analysis After Shooting Laser

When the exposure time is beyond 30 seconds, the 3D printed substrate got damaged so a constant time of 10 seconds has been maintained throughout the study.

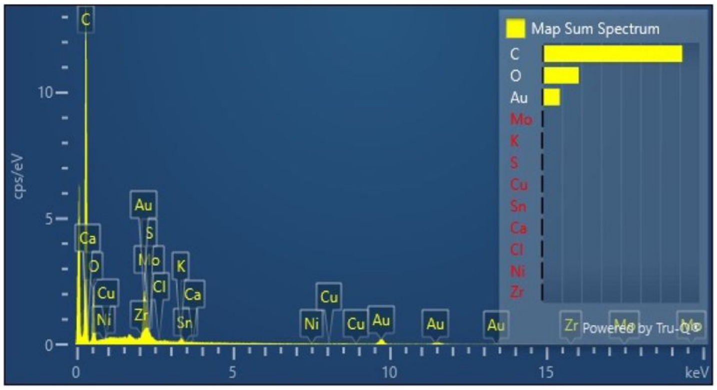

Figure S3. EDS spectrum depicting the spatial distribution of (Carbon (C), Oxygen (O), and Gold (Au) elements after the laser.

4. Deformed 3D Printed Substrate when exposed to laser

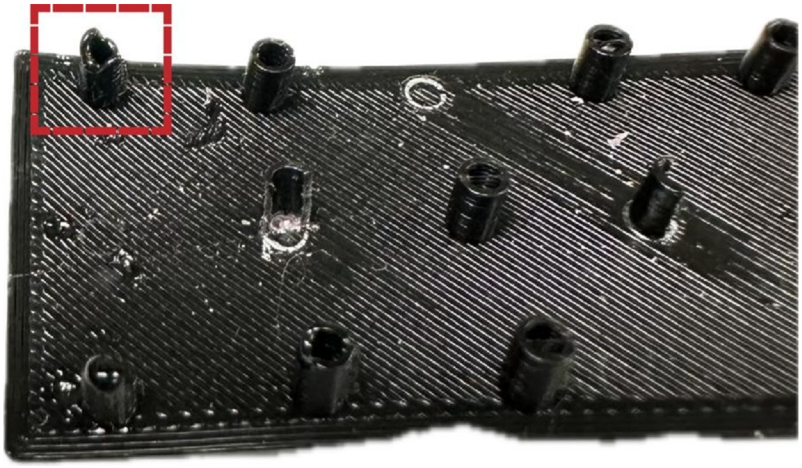

Figure S4. Laser effect on 3D printed substrate when the power density 40.74 W/cm^2 exposed for 30 seconds.

5. EDS Analysis in the presence of carbon coating

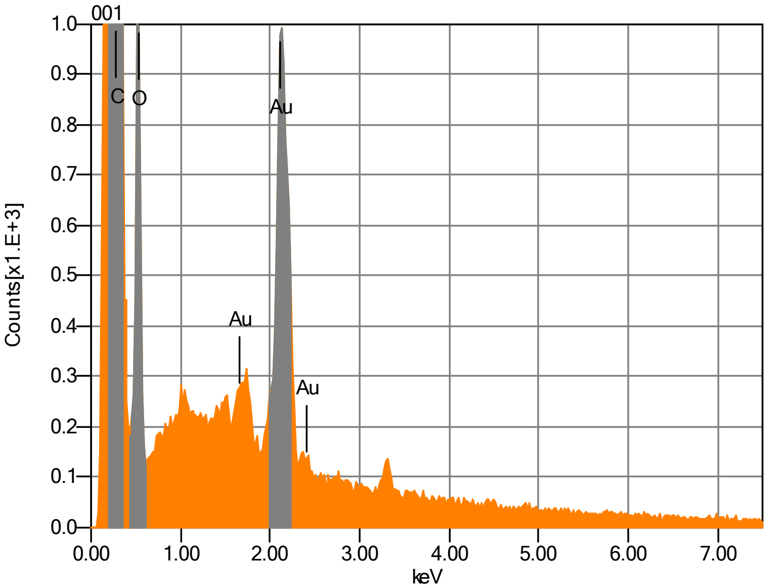

Figure S5. EDS images illustrating the spatial distribution of (Carbon (C), Oxygen (O), and Gold (Au) elements in the presence of carbon coating.

6. Thermophoretic Force and Discussion

Thermophoretic force[1] is produced when nanoparticles in heat gradient move from regions of high temperatures to low, described as below:

$$\text{Thermophoretic Force} = \frac{6\pi d_p \mu^2 C_s \Lambda \nabla T}{\rho(2\Lambda+1)T} \quad (1)$$

Where $\Lambda = k/k_p$, k is the thermal conductivity of the fluid, k_p is the particle thermal conductivity, T is the fluid temperature, ∇T is heat gradient, d_p is the particle diameter, μ is the fluid dynamic viscosity, ρ is the fluid density, C_s is a dimensionless constant equal to 1.17. In our case, AuNPs are illuminated by 532 nm laser and produce heat gradient around AuNPs environment due to photothermal effect. At hot zone, molecules are heated and move more actively compared that at cold zone, which exerts pressure on AuNPs and cause movement along heat gradient. When laser power is low at 6.76 W/cm^2, the heat gradient is small and solution viscosity and density is high so that the thermophoretic force is low, which only assembles AuNPs around small regions. When laser power is increased to 13.45 W/cm^2, the heat gradient is larger. As a result, solution viscosity and density decrease meaning that the thermophoretic force increases, assembling AuNPs along larger areas to form nanochains. When further increasing laser power to 26.89 W/cm^2, the heat gradient is further enhanced, and solution viscosity density is even lower around a larger area, which exerts a force on AuNPs around a larger area. Due to the Gaussian beam shape, the laser decays from center to edge so that the nano-chains are formed into nanonetwork along the beam.

7. Literature survey on self-assembly of AuNPs via different 3D Printing Techniques

Table S1. Literature reviews on AuNPs assembly via different 3D printing techniques

3D Printing Technique	Assembly Mechanism	Pattern Morphology	Reference
Two-photon-polymerisation	electrodeposition	Conical cavities	2
Electrohydrodynamic-jet printing	electrostatic assembly	Linear Patterns	3
Electrohydrodynamic printing technique	electrostatic nanodroplet autofocussing	Nanopillars printed subsequently at 200 nm center-to-center distance, 80-nm wide dots printed into a 1-µm lattice constant array	4
Electrohydrodynamic-jet printing	Electrohydrodynamic nano-drip printing mode	Gold grids with line width of 80-500nm	5
Direct Ink Writing	Alloying and dealloying	hierarchical nano-porous /ligament morphology (30 to 500 nm)	6
Micronozzle 3D printing	Evaporative self-assembly	Hierarchical plasmonic clusters	7
**Fused Deposition Modeling with ink dropping**	**Plasmonic light-driven self-assembly**	**Nanoparticle Networks with tunable morphologies**	**Our work**

7. References

(1) Loyalka, S. K. Thermophoretic Force on a Single Particle—I. Numerical Solution of the Linearized Boltzmann Equation. *J Aerosol Sci* **1992**, *23* (3), 291–300, DOI: 10.1016/0021-8502(92)90329-T.

(2) Šakalys, R.; Kho, K. W.; Keyes, T. E. A Reproducible, Low Cost Microfluidic Microcavity Array SERS Platform Prepared by Soft Lithography from a 2 Photon 3D Printed Template. *Sens Actuators B Chem* **2021**, *340*, DOI: 10.1016/j.snb.2021.129970.

(3) Porter, B. F.; Mkhize, N.; Bhaskaran, H. Nanoparticle Assembly Enabled by EHD-Printed Monolayers. *Microsyst Nanoeng* **2017**, *3* (1), 17054, DOI: 10.1038/micronano.2017.54.

(4) Galliker, P.; Schneider, J.; Eghlidi, H.; Kress, S.; Sandoghdar, V.; Poulikakos, D. Direct Printing of Nanostructures by Electrostatic Autofocussing of Ink Nanodroplets. *Nat Commun* **2012**, *3* (1), 890, DOI: 10.1038/ncomms1891.

(5) Schneider, J.; Rohner, P.; Thureja, D.; Schmid, M.; Galliker, P.; Poulikakos, D. Electrohydrodynamic NanoDrip Printing of High Aspect Ratio Metal Grid Transparent Electrodes. *Adv Funct Mater* **2016**, *26* (6), 833–840, DOI: 10.1002/adfm.201503705.

(6) Zhu, C.; Qi, Z.; Beck, V. A.; Luneau, M.; Lattimer, J.; Chen, W.; Worsley, M. A.; Ye, J.; Duoss, E. B.; Spadaccini, C. M.; Friend, C. M.; Biener, J. Toward Digitally Controlled Catalyst Architectures: Hierarchical Nanoporous Gold via 3D Printing. *Sci Adv* **2024**, *4* (8), eaas9459, DOI: 10.1126/sciadv.aas9459.

(7) Kim, G.; Lee, J.; Devaraj, V.; Kim, M.; Jeong, H.; Choi, E. J.; Yang, J.; Lee, D.; Kim, J. T.; Oh, J.-W. Freeform 3D Plasmonic Superstructures, DOI: 10.21203/rs.3.rs-68346/v1.
